# Guidance for contact tracing of cases of Lassa fever, Ebola or Marburg haemorrhagic fever on an airplane: results of a European expert consultation

**DOI:** 10.1186/1471-2458-12-1014

**Published:** 2012-11-21

**Authors:** Andreas Gilsdorf, Dilys Morgan, Katrin Leitmeyer

**Affiliations:** 1 Department for Infectious Disease Epidemiology, Robert Koch Institute, Berlin, Germany; 2 Health Protection Agency, London, United Kingdom; 3 European Centre for Diseases Prevention and Control, Stockholm, Sweden

**Keywords:** Viral haemorrhagic fever, Lassa fever, Ebola haemorrhagic fever, Marburg haemorrhagic fever, Passenger trace back, Contact tracing, Air travel

## Abstract

**Background:**

Travel from countries where viral haemorrhagic fevers (VHF) are endemic has increased significantly over the past decades. In several reported VHF events on airplanes, passenger trace back was initiated but the scale of the trace back differed considerably. The absence of guidance documents to help the decision on necessity and scale of the trace back contributed to this variation.

This article outlines the recommendations of an expert panel on Lassa fever, Ebola and Marburg haemorrhagic fever to the wider scientific community in order to advise the relevant stakeholders in the decision and scale of a possible passenger trace back.

**Method:**

The evidence was collected through review of published literature and through the views of an expert panel. The guidance was agreed by consensus.

**Results:**

Only a few events of VHF cases during air travel are reported in literature, with no documented infection in followed up contacts, so that no evidence of transmission of VHF during air travel exists to date. Based on this and the expert opinion, it was recommended that passenger trace back was undertaken only if: the index case had symptoms during the flight; the flight was within 21 days after detection of the event; and for Lassa fever if exposure of body fluid has been reported. The trace back should only be done after confirmation of the index case. Passengers and crew with direct contact, seat neighbours (+/− 1 seat), crew and cleaning personal of the section of the index case should be included in the trace back.

**Conclusion:**

No evidence has been found for the transmission of VHF in airplanes. This information should be taken into account, when a trace back decision has to be taken, because such a measure produces an enormous work load. The procedure suggested by the expert group can guide decisions made in future events, where a patient with suspected VHF infection travelled on a plane. However, the actual decision on start and scale of a trace back always lies in the hands of the responsible people taking all relevant information into account.

## Background

In recent years, with increasing numbers of passengers travelling internationally by air the potential risk of introduction and spread of rare infectious diseases by travellers has increased.

In 2010, 5.04 billion passengers arrived and departed from 1318 airports worldwide, nearly half of them on international flights [1], and travel to and from Africa almost doubled between 1995 and 2005 [2]. The number of tourists reaching more remote areas and risking exposure to viral haemorrhagic fever (VHF) viruses has also increased. This has contributed to an increase of reported events in which a passenger with a VHF travelled on board an aircraft. Although the air transportation of a passenger suffering from a VHF is still rare, and has led to reported secondary transmission in the country of destination only once [3], the severe potential outcome of the disease and the public perception of its infectiousness result sometimes in high media attention. Often this public pressure influences the decision on public health measures, such as passenger trace back, more than the existing evidence. In several reported VHF events on airplanes, passenger trace back was initiated [4] but the scale of the trace back response differed considerably. Absence of guidance documents to help the decision on necessity and scale of the trace back may well have contributed to this variation.

In order to assist national public health authorities in European Union (EU) Member States to evaluate the risks related to the transmission of a VHF on board of aircrafts and to help in the decision on the most appropriate, operationally possible, public health measures for containment, the European Centre for Disease Prevention and Control (ECDC) initiated in 2007 the project “Risk assessment guidance for infectious diseases transmitted on aircraft” (RAGIDA) [5].

The RAGIDA project consisted of two parts I) a systematic review of the literature of documented events of infectious disease transmission on aircrafts, guidance documents and expert interviews assessing case-based information on events [6], and II) a series of disease-specific guidance documents produced by external expert panels based on the literature review and their personal expertise [7]. This article reports the recommendations of the expert panel on the VHFs – Lassa fever, Ebola and Marburg haemorrhagic fever that are included in the ECDC guidance document [7]. It aims to reach and advise the wider scientific and public health community and other relevant stakeholders on the necessity to implement a passenger trace back and the scale of the response.

## Methods

The second part of the RAGIDA project initiated the production of a series of operational guidance documents for assisting in the evaluation of risk for transmission of diseases. As for all other disease groups within RAGIDA, a small, multidisciplinary expert group meeting was held in June 2010 to consider Lassa fever, Ebola and Marburg haemorrhagic fever. These particular VHFs had been reported in context of past passenger trace back activities. The participants were selected to include: representatives of national public health authorities with experience in the investigation and follow-up of incidents involving VHFs in travellers; European and international disease experts; representatives of ECDC and of the World Health Organizations Regional Office for Europe (WHO-EURO). All participants completed a declaration of interest form. No conflicts of interest were declared by any of the participants. The participants are listed in the acknowledgement of this article.

The evidence collected through the review of scientifically published and grey literature in part I of the RAGIDA project, was considered by the participants [6]. In addition, evidence was provided based on experiences and opinions of the expert panel. In developing the guidance, not only the available scientific evidence for disease transmission were taken into account, but also wider aspects including disease severity, the potential for public health intervention and availability of treatment. A draft approach was discussed and agreed on at the meeting. The final guidance document of the expert panel was agreed by consensus and validated by the members of the ECDC Advisory Forum in September 2010.

## Results

### Literature review

#### Lassa fever

A detailed systematic literature review identified nine incidents of Lassa fever cases being imported into Europe, (including one case which was in transit in London while travelling to the U.S.) between 2000 and 2010 [8-17].

Details about contact tracing were available for seven of the events. Contact tracing was initiated in all seven events because the index cases were symptomatic on-board, and the incubation period still allowed for preventive measures to be taken. In two events, a comprehensive follow up was initiated, and passengers could be traced because their seat location in relation to the index case’s seat was known. Contact tracing was done by actively contacting passengers with the help of airline manifests, 179/293 contacts were successfully traced, and none developed the disease [6].

The literature review showed that existing evidence suggested a low risk of transmission of Lassa fever during air travel, it also suggests that the risk remains low even if a high risk exposure occurred [9,12].

#### Ebola haemorrhagic fever

The literature review only found one article in the peer-reviewed journals related to Ebola virus on a flight [18], but this was a repatriation flight of the patient who was already very sick. Only 4 crew members were in contact with the patient, and neither the crew nor any of the other 74 contacts identified and tested, showed evidence of sero-conversion. One article was retrieved from the grey literature that reported a patient who took a commercial flight from Gabon to Johannesburg in 1996 for hospital treatment [3]. During the flight, the diagnosis of Ebola haemorrhagic fever (later laboratory confirmed) was not known. He presented with fever and jaundice, both not severe. He was only traced back after a nurse caring for him died and Ebola haemorrhagic fever was diagnosed. This happened sometime after the flight, so no passenger trace back was initiated.

With this low number of events of Ebola haemorrhagic fever on flights, other studies describing transmission risk of Ebola haemorrhagic fever were examined to describe the likelihood of transmission. The reviewed studies show a low risk of transmission in the early phase of symptomatic patients, even if high risk exposure occurred. However, risk of transmission may increase in later stages of the disease with increasing viral titres [19] and increased viral shedding. In a household study, secondary transmission only took place if direct physical contact occurred [20]; In an outbreak in 2000 in Uganda, the most important risk factor was direct and repeated contact with a sick person’s body fluids, as occurs during the provision of care. The risk was higher when the exposure took place during the late stage of the disease. However, one case was probably infected by contact with heavily contaminated fomites, and many persons who had had a simple physical contact with a sick person did not become infected. Therefore transmission through heavily contaminated fomites is apparently possible [21]. In summary, physical contact with body fluids seems necessary for transmission, especially in the early stages of disease (as is likely in passengers still able to travel on a plane), while in the later stages contact with heavily contaminated fomites might also be a risk for transmission.

#### Marburg haemorrhagic fever

The literature review for Marburg haemorrhagic fever showed few peer reviewed reports. One was an event where a Marburg haemorrhagic fever patient travelled on a plane to the Netherlands in July 2008 [22]. No transmission occurred in the followed up passengers in this event. A U.S. tourist who had visited the same bat cave in Uganda as the Dutch case in January 2008, developed symptoms after returning to the U.S. and was retrospectively diagnosed in January 2009, none of the 260 identified contacts developed severe febrile illness [23].

Published information about risk of transmission is very sparse. The WHO fact sheet on Marburg haemorrhagic fever states that transmission of the virus from person to person requires extremely close contact with a patient. Infection results from contact with blood or other body fluids (faeces, vomitus, urine, saliva, and respiratory secretions) with high virus concentration, especially when these fluids contain blood. Infection through casual contact is thought to be exceedingly rare [24,25]. The largest Marburg haemorrhagic fever outbreak recorded was in Angola in 2005, with 374 reported cases (158 laboratory confirmed) and 329 deaths [26]. The disease spread particularly among people exposed to Marburg virus during home care or at funerals, via contact with body fluids of those who died from the disease. The dangerous use of home-based injections was also identified as a major cause of the outbreak’s spread [27,28].

However, in a study in the Democratic Republic of Congo in 1999, no antibodies were found in health care workers despite frequent high risk procedures [25]. In the first reported outbreak in 1967, the 32 cases reported produced only 6 secondary infections in close family members [29-31]. In another study only 1 of 207 close contacts of a case patient with Marburg haemorrhagic fever contracted the virus [32].

### Guidance

Based on the literature reviews the expert group developed a trace back guidance by disease. The risk assessment of possible transmission of VHF on an aircraft should be undertaken on a case-by-case basis. This should take into account information on the index case status, the epidemic situation of the country where the index case most likely acquired the infection, the possible exposure of the index case and how long the event has been detected after the flight. When to consider a passenger as probable case, was based on symptom description by WHO and probable exposure to a source of the respective VHF. The decision guidance is summarised by disease in Table 1.

**Table 1:** Risk assessment for trace back of patients with a Viral Haemorrhagic Fever (Lassa fever, Marburg, or Ebola haemorrhagic fever), who travelled on an airplane

	**Lassa**	**Ebola**	**Marburg**
**Index case**	**Probable or laboratory confirmed cases can be considered for trace back**		
	A patient could be considered as a probable case of Lassa	A patient could be considered as a probable case of Ebola	A patient could be considered as a probable case of Marburg
	1. Who has symptoms compatible with Lassa (malaise, fever, headache, sore throat, cough, nausea, vomiting, diarrhoea, myalgia, chest pain, hearing loss [33]) AND	1. Who has symptoms compatible with Ebola (sudden onset of fever, intense weakness, muscle pain, headache, sore throat, vomiting, diarrhoea, rash, impaired kidney and liver function, internal and external bleeding [34] AND	1. Who has symptoms compatible with Marburg (abrupt onset, severe headache, severe malaise, muscle aches and pains, high fever, severe watery diarrhoea, abdominal pain and cramping, nausea, vomiting [24]) AND
	2. Who had within 21 days of symptom onset	2. Who had within 21 days of symptom onset	2. Who had within 21 days of symptom onset
	a) Risk exposure to rats or their droppings in rural areas in West Africa [35] OR	a) Risk exposure in Sub-Saharan Africa (medical treatment, contact to body fluids of ill persons, contact with primates or bats in areas with suspected or known Ebola activity [36]) OR	a) Risk exposure in Sub-Saharan Africa (medical treatment, contact with body fluids of ill persons, contact with primates or bats. in areas with suspected or known Marburg activity [36]) OR
	b) Contact to a case of Lassa (e.g. health care worker, care giver, etc.)	b) Contact with a case of Ebola.	b) Contact with a case of Marburg.
	WHO recommends a case definition for surveillance standards that could be also helpful for contact tracing [37].		
**Epidemic situation**	**Travel to West Africa**	**Travel to Sub-Saharan Africa**	
	Certain West African countries are considered endemic areas for transmission of Lassa fever [35]. However, non-endemic countries may also be taken into consideration if the passenger has particular risk exposures. Although the epidemiology remains to be determined, a new Arenavirus called Lujo, which is similar to Lassa virus, has been identified in case from Zambia with secondary transmission in South Africa [38].	Certain Sub-Saharan African countries are considered as risk areas for transmission [36]. However not only those African countries where already cases have been reported should be taken into consideration, as the index patient could be the first case to indicate infection in a country.	Certain Sub-Saharan African countries are considered as risk areas for transmission [36]. However not only those African countries where already cases have been reported should be taken into consideration, as the index patient could be the first case to indicate infection in a country.
	Risk exposure: The reservoir of Lassa virus is a rodent host *M.natalensis*, in which it is persistent and mostly silent. [39] Outbreaks have also been reported in hospital settings [40].	Risk exposure: Evidence indicates to bats as one of the reservoir of Ebola [41]. On the African continent, Ebola infections of human cases have been linked to direct contact with gorillas, chimpanzees, monkeys, forest antelope and porcupines found dead in the rainforest. [34,42,43]. Human-to-Human transmission has taken place during medical treatment, through direct contact with body fluids of ill or dead persons. Outbreaks have also been reported in hospital settings. This should be taken into consideration when assessing the risk exposure of a probable case.	Risk exposure: Evidence indicates to bats as one of the reservoir of Marburg [44,45]. Human-to-Human transmission route is through direct contact with blood or other infected body fluids. Outbreaks have also been reported in hospital settings. This should be taken into consideration when assessing the risk exposure to a probable case.
**Effective Exposure**	**Direct contact to body fluids**	**Direct contact with case even if exposure to body fluids was not reported**	
	Contact tracing of a Lassa case, should only be considered if direct contact to body fluids such as blood, urine, faeces or vomit had taken place during the flight. Unless such an incident took place, the likelihood of a transmission is considered negligible.	Human-to-human transmission of Ebola virus occurs through direct contact with infectious body fluids. However, Ebola virus has also been detected in sweat [46], and although the risk is very low, passengers who may have had direct contact with the case should be contacted and followed-up, even if exposure to body fluids was not reported.	Human-to-human transmission of Marburg virus occurs through direct contact with infected body fluids. As the transmission of Marburg virus through sweat cannot be excluded, and although the risk is very low, passengers who may have had direct contact with the case should be contacted and followed-up, even if exposure to body fluids was not reported.
**Timing of flight**	**Detection of the event within 21 days after the flight**		
	The incubation period of Lassa is usually seven to 12 days but may range between three and 21 days [32,47-49]. In order to find potential cases, tracing passengers should only be considered if the flight took place within the previous 21 days.	The incubation period of Ebola usually ranges between two and 21 days [50]. Thus in order to find potential cases, tracing passengers should only be considered if the flight happened within the previous 21 days.	Incubation period for Marburg ranges between 2 and 14 days [51]. In order to find potential cases within the possible longest incubation period, tracing passengers should only be considered, if the flight happened within the previous 21 days. To have a common approach with the other VHF it was decided to use for Marburg also the 21 days period.
	After this time period a message to raise awareness among doctors and public health professionals should be considered.	After this time period, a message to raise awareness among doctors and public health professionals could be considered.	After this time period a message to raise awareness among doctors and public health professionals could be considered.

**Figure 1:**
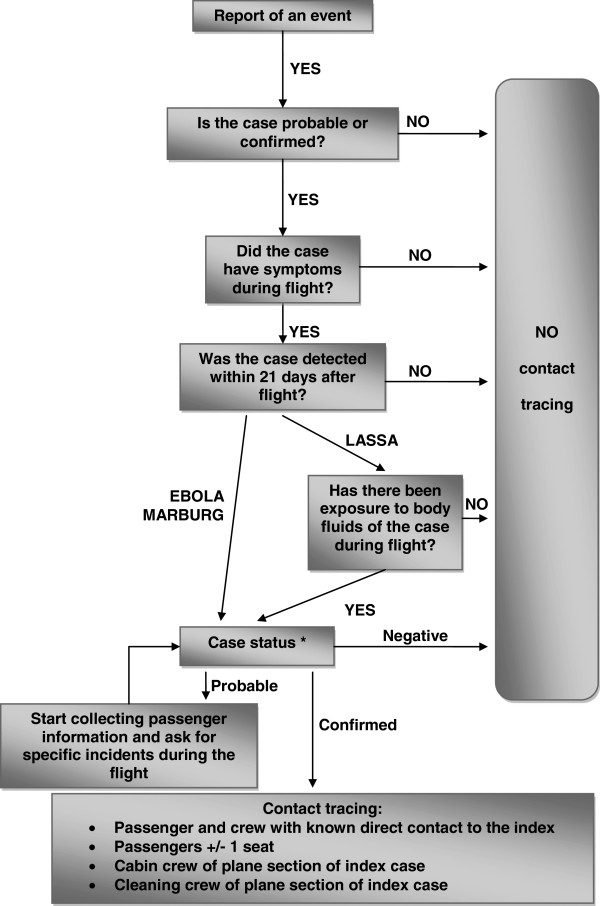
**Risk assessment algorithm: Viral Haemorrhagic Fever (Lassa fever, Marburg or Ebola haemorrhagic fever).** * If the diagnosis cannot be laboratory confirmed (e.g. if clinical samples are unavailable), contact tracing should be considered if the clinical and epidemiological picture is strongly suggestive of a VHF as the likely diagnosis.

The decisions for initiating a trace back are outlined in the risk assessment algorithm in Figure 1.

Since direct contact is the main route of transmission for VHF, the trace back should be mainly limited to passengers and crew who were close to the index case. The following should be included in the trace back:

Passengers and crew with reported direct contact

Co-travellers and crew members who had reported direct body contact to the index case should be traced. To gather this information, any records of significant events on the flight should be obtained from the airline

Passengers +/−1 seat

As direct contact is the main route of transmission for the three VHFs, only the passengers who sat in direct proximity to the index passenger should be included into the trace back. That means only passengers, who sat one seat in all four directions from the index case in all directions should be traced backed. If the index case sat on an aisle seat, also the passengers sitting one seat across the aisle should be contacted.

Crew members of plane section

Crew members who served in the section of the index case should be included in the trace back, as well as any other crew members who had direct with the patient e.g. they had assisted him or her.

Cleaning staff of plane section

The cleaning staff who were responsible for cleaning the section and seat where the index case sat should be traced back, and assessed as to whether effective personal protective equipment had been used.

## Discussion

The usual methodology for producing guidance is based on the assessment of scientific evidence mainly from literature reviews and expert opinion. However, because of the lack of relevant publications regarding a patient with a VHF on flight, the expert opinion approach was chosen. The group participants represented a wide range of expertise and experience. Beside the risk of transmission, other facts have to be considered. Even though there is no specific treatment available for Marburg and Ebola infections, early supportive care should improve the outcome of cases. Also, reason of starting contact tracing should be to raise awareness and prevent onward transmission. Specific treatment is available for Lassa virus infection, but this is most effective if initiated early in the disease, so initiation of antiviral treatment is an additional reason to consider a trace back [52].

Patients with more severe symptoms are more infectious, but as it is difficult to judge when the symptoms indicate infectiousness, severity was not considered as a criterion to decide for trace back but only the presence of any symptoms compatible with VHF during the flight.

Cases of Lassa fever, Ebola or Marburg haemorrhagic fever were not considered to be infectious before they developed symptoms [53]. Therefore a trace back should only be initiated, if the index patient was symptomatic on board. These would include non-specific symptoms, as these may occur in the early stages of infection.

The main route of transmission for a VHF infection is by direct contact with infectious body fluids. The transmission of a VHF through aerosol spread was considered as negligible. In the absence of specific incidents involving body fluids, the use of a toilet by the index case is not considered as a risk factor and therefore would not be considered in the contact tracing. Since direct contact is necessary for the transmission of a VHF, the duration of flight is not taken into consideration for the decision to start a trace back. We recommend trace back to be initiated following laboratory confirmation of the diagnosis. However, the airline should be contacted to enquire whether crew members remembered or recorded any incidents on board which might have resulted in potential exposures to crew or passengers and the availability of the passenger manifest while awaiting the laboratory result. This will facilitate prompt actions should a VHF be confirmed. If a diagnosis cannot be laboratory confirmed in a timely manner, contact tracing should be considered if evidence strongly suggests a VHF as the likely cause of disease in the index case.

## Conclusions

Only few events of VHF cases during air travel have been reported in literature, with no documented transmission in contacts who were followed up. Hence, evidence for transmission is lacking. Therefore, the expert group considered the risk of transmission of a VHF from an infected patient during a flight to be very low. This information should be taken into account when a trace back decision has to be taken, because such a measure produces a significant workload for many people involved. The procedure suggested by the expert group will guide decision takers in future events where a patient with a suspected VHF infection travelled on a plane. However, the actual decision on start and scale of a trace back always lies in the hands of the responsible people taking all relevant information into account.

## Competing interests

The authors have no competing interests.

## Authors’ contributions

AG, DM and KL were all participants in the RAGIDA VHF expert group. AG has been the reporter during the expert meeting, mainly prepared the report of the meeting and he has written this article and organised the review by the group. DM has chaired the expert meeting, and contributed significantly both to the report and article. KL organised the expert meeting and contributed hugely to the report and article. All authors read and approved the final manuscript.

## Pre-publication history

The pre-publication history for this paper can be accessed here:

http://www.biomedcentral.com/1471-2458/12/1014/prepub
